# Antimicrobial effects and mechanical properties of poly(methyl methacrylate) as an orthodontic acrylic resin containing Curcumin-Nisin-poly(l-lactic acid) nanoparticle: an in vitro study

**DOI:** 10.1186/s12903-022-02197-z

**Published:** 2022-05-06

**Authors:** Maryam Pourhajibagher, Mohammad Noroozian, Mohammad Sadegh Ahmad Akhoundi, Abbas Bahador

**Affiliations:** 1 Dental Research Center, Dentistry Research Institute, Tehran University of Medical Sciences, Tehran, Iran; 2 Department of Orthodontics, School of Dentistry, Ilam University of Medical Sciences, Ilam, Iran; 3 Student Research Committee, School of Dentistry, Ilam University of Medical Sciences, Ilam, Iran; 4 Department of Orthodontics, School of Dentistry, Tehran University of Medical Sciences, Tehran, Iran; 5 Fellowship in Clinical Laboratory Sciences, BioHealth Lab, Tehran, Iran

**Keywords:** Curcumin, Nisin, Biofilms, Orthodontic acrylic resin

## Abstract

**Background:**

The porous surface of acrylic orthodontic removable appliances creates a niche for microbial plaque accumulation, and changes the oral flora by raising cariogenic bacteria including *Streptococcus mutans*. In this study, we evaluated the mechanical properties and antimicrobial activities of incorporating different concentrations of Curcumin-Nisin-poly(l-lactic acid) nanoparticle (CurNisNps) into orthodontic acrylic resin against *Streptococcus mutans* and *Candida albicans*.

**Methods:**

Following synthesis and characterization of CurNisNps, acrylic resin specimens with different concentrations of CurNisNps (0, 1, 2, 5, and 10% w/w) were fabricated. Flexural strength values, antimicrobial effects, anti-biofilm potential, and anti-metabolic activity against *S. mutans* and *C. albicans* were assessed at different time intervals. Also, the expression of the virulence-factor-related genes of *S. mutans* and *C. albicans* was assessed by quantitative real-time polymerase chain reaction following treatment with CurNisNps.

**Results:**

Acrylic resin containing 10% CurNisNps (30.76 ± 3.91 MPa) showed flexural failure in comparison with acrylic resin specimens without CurNisNps (50.67 ± 1.82 MPa) as the control group (*P* < 0.05). There was no significant decrease in the flexural strength values in samples containing 1, 2, and 5% of CurNisNps in comparison to the control group (*P* > 0.05). Acrylic resin with 5% CurNisNps showed the highest concentration of CurNisNps and clinically accepted flexural strength value (14.89 ± 3.26 MPa, *P* < 0.05) simultaneously. In the disc agar diffusion assay, 5% CurNisNps showed a high level of inhibitory activity for the test microorganisms. The reduction of growth inhibition zones of the different concentrations of CurNisNps against test microorganisms was positively associated with the time, in such a way that it was reduced significantly after 60 days. The anti-biofilm and anti-metabolic activities of acrylic resin specimens containing a 5% concentration of CurNisNps against *S. mutans* and *C. albicans* could significantly decrease the expression levels of *gtfB* (6.8-fold) and *HWP* (3.4-fold) in *S. mutans* and *C. albicans*, respectively.

**Conclusions:**

Our data support that 5% (w/w) of CurNisNps can serve as an excellent orthodontic acrylic resin additive against *S. mutans* and *C. albicans* biofilm without adverse effects on its mechanical property.

## Background

Orthodontic acrylic removable appliances based on poly(methyl methacrylate) (PMMA) are commonly used for minor tooth movement, growth modification treatments in children, and as a retainer after the end of fixed orthodontic treatment [1]. These appliances are made from cold cure acrylic resins which have more porosity in their structure than heat cure ones that are usually used for fabricating removable dental prosthesis [2]. The porous surface of orthodontic removable appliances creates a niche for microorganisms; causes more plaque colonization, and changes the oral flora by raising cariogenic bacteria such as *Streptococcus mutans* and lactobacilli [3, 4]. Formation of the bacterial and fungal (specially *Candida albicans*) biofilms surrounding these appliances due to their irregular surfaces and limitation of the mechanical self-cleaning process providing by saliva results in some complications such as dental caries, periodontal diseases, and candidiasis in susceptible patients [5, 6].

Mechanical cleaning of removable acrylic appliances is beneficial in decreasing biofilm accumulation, however, could not eliminate all microorganisms as they can penetrate into the acrylic base as deep as 1 to 2 mm [7]. Immersion in antimicrobial solutions including chlorhexidine, NitrAdine, or cetylpyridinium is another way for cleaning them but the possibility of damage to surface integrity following frequent use has been reported [8, 9]. Moreover, such measures depend primarily on a patient’s compliance and this cleaning process may not be performed properly in children who use more of these devices. Dental plaque consists of various microorganisms that are surrounded by a matrix of polymers originating from bacteria and host. Biofilm development happens in several stages. Glycosyltransferase (GTF), a streptococcal enzyme that converts glucose to glucan polymers, plays a major role in the formation of dental plaque. This viscose polysaccharide facilitates the binding and accumulation of cariogenic bacteria. *S. mutans* is a pivotal microorganism that takes part in the progression of dental caries due to its high acid production capacity, and so it has been used to evaluate the antimicrobial properties of dental materials in previous experimental studies [10]. Hyphal wall protein (Hwp), a transglutaminase substrate which functions as an adhesin, is important for the pathogenesis of candidiasis. The previous study reported the *HWP* gene expression implicates the ability of *C. albicans* to establish and maintain its presence on oral mucosal surfaces of human hosts [11].

Dental biofilm is considered by high pathogenicity and low susceptibility to antimicrobial agents since the structure of the biofilm may inhibit the penetration of antimicrobial agents. As a result, preventing the formation of biofilm on the surface of acrylic appliances is more reasonable than removing it later [11, 12].

Many studies have been conducted to assess the self-sterilizing properties of modified acrylic resins material. Different nanoparticles such as TiO_2_ [13], silver [14], ZnO [15], and platinum [16] have been combined with acrylic resins to give it self-sterilizing feature, however, the biocompatibility of these newly synthesized acrylic resins may be negatively affected by the potential releasing of metal ions in the oral environment during a long period of use [17, 18].

Curcumin (Cur) has antimicrobial, antioxidant, and anti-inflammatory properties [19]. It hinders acid production by *S. mutans*, and can remarkably inhibit the adhesion of *S. mutans* to surfaces [20]. Cur-mediated antimicrobial photodynamic therapy (aPDT) has shown significant antibacterial effects [21]. Cur mouthwash is as effective as chlorhexidine, the gold standard antimicrobial mouthwash [22]. Pulp capping agent doped with Cur has significant antimicrobial activity against *S. mutans*, thus can prevent secondary caries under dental restorations [23]. Using Cur in the form of nanoparticles improves its bioavailability, reduces its decomposition rate, increases its stability in blood flow, and reduces its cytotoxicity [24].

Nisin (Nis) is a natural antibacterial agent that is produced by *Lactococcus lactis* and has been approved by the World Health Organization (WHO) as a safe preservative in food industries [25]. Nis inhibits the growth of cariogenic bacteria in the planktonic form at low concentrations (2.5–0.5 μg/mL) and can hinder the development of multi-species biofilm at concentrations higher than 1 μg/mL. Nis degrades the biofilm structure and can reduce its volume and thickness in a dose- and time-dependent manner. Also, it reduces the number of viable bacteria in the biofilm composition. In addition, anti-biofilm concentrations of Nis do not have any toxic effect on human oral cells [25].

Autopolymerizing cold cure acrylic resins are commonly used for manufacturing orthodontic acrylic appliances due to their easy manipulation but they still have certain poor mechanical characteristics and are susceptible to fracture during clinical application. Among diverse mechanical properties, flexural strength has received the most attention in previous studies. So, it is highly suggested to investigate the impact of any additive on their mechanical properties to avoid the harmful effects that might reduce their flexural strength below the standard level [26].

The first aim of this study is to determine the optimal concentration of Cur-Nis-poly(l-lactic acid) nanoparticle (CurNisNps) as an additive to acrylic resin in a way that does not significantly affect its mechanical properties. The second aim of the present study is to evaluate the antimicrobial and anti-biofilm properties, as well as, anti-virulence effects of this new synthetic compound against common cariogenic microorganisms (*S. mutans* and *Candida albicans*).

## Methods

### CurNisNps preparation

CurNisNps were prepared by the double emulsion-diffusion-evaporation method [27]. Briefly, an aqueous phase was prepared by dissolving 5 mg of curcumin (0.092 g; 2.5 mmol) and 5 mg of Nisin in 200 µL polyvinyl alcohol (PVA; All purchased from Sigma-Aldrich, United Kingdom). An organic phase was provided by dissolving 50 mg of poly-lactic acid (PLA; Merck, Germany) in 3.5 mL of dichloromethane and 0.5 mL of acetone (Both obtained from Sigma-Aldrich, United Kingdom). The aqueous phase was dispersed in a drop-wise way into the organic solution and then was sonicated continuously for one minute to form the initial emulsion. The emulsion was then added to another aqueous phase containing 16 mL PVA and 1 wt% sucrose and was sonicated continuously for 3 min to form the final suspension. The final suspension was magnetically stirred at room temperature for 6 h to form a colloidal suspension of CurNisNps. Eventually, the suspension was centrifuged at 16,000 rpm for 15 min and the obtained sediment was washed with distilled water three times. The final product was transferred to a freeze-dryer to obtain a dry powder.

### CurNisNps characterization

The surface morphology of CurNisNps was studied by field emission scanning electron microscopy (FESEM; ZEISS, German) according to the E2578-07(2018) [28] and ISO 16,700:2016 [29] guidelines. The size distribution profiles of nanometer-sized particles and zeta potential of the CurNisNps were carried out using a MALVERN Zetasizer Ver. 6.01 (Malvern Instruments, UK) at approximately 25 °C based on the guidance of E2865-12(2018) [30]. Also, fourier-transform infrared (FTIR) analysis was performed using a spectrum of two spectrophotometers (45° ZnSe crystal, PerkinElmer Inc., US), within the range of 1000–4000 cm^−1^ according to the E1252-98(2021) guideline [31].

### Sample size

In the present study, the test groups including modified poly(methyl methacrylate) (PMMA) containing different concentrations (1, 2, 5, and 10%) of CurNisNps and PMMA alone as the control group were assessed. For the flexural strength test, the sample size was calculated based on the previous study [26] and by using G * Power software (Version 3.1.9.2; http://gpower.hhu.de/). The sample size for this test was determined 10 in each of the 5 groups (control, 1, 2, 5, 10%). The sample size for antimicrobial tests was calculated by using the One-Way ANOVA Power Analysis formula and was determined 4 in each group.

### Specimen preparation

The acrylic resin sticks with 0, 1, 2, 5, and 10% (w/w) concentrations of CurNisNps were fabricated for flexural strength test based on the previous study [32].

### Flexural strength test

A universal testing machine (ZWICK Z250, Zwick Roell Group, Herefordshire, UK) was used for the flexural strength test. The amount of flexural strength in MPa was calculated based on the formula below:

$$\sigma =\frac{3.f.I}{2.b.{h}^{2}}$$

F = The maximum applied force in Newton, I = The distance across the supporter arms of machine measured in mm, b = The width of specimens in mm, h = The height of specimens in mm.

### Artificial aging on acrylic discs containing selected CurNisNps

Before the anti-biofilm test, the acrylic discs were stored in artificial saliva (0.2 g NaCl, 0.2 g KCl, 0.453 g CaCl_2_.2H_2_O, 0.345 g NaH_2_PO_4_.2H_2_O, 0.5 g urea in 1000 mL of DI water; pH of 7) with 7.5 ± 0.5 mL/h flow rate at 37 °C until 60 days. Four discs from each group were used to evaluate the anti-biofilm activity of the discs at 0, 15, 30, and 60 days.

### Microbial strains and culture conditions

The standard strain of *S. mutans* ATCC 35668 and *C. albicans* ATCC 14053 were provided from the Iranian Biological Resource Center, Tehran, Iran. *S. mutans* was cultured in brain heart infusion (BHI) broth (Merck, Germany) at 37 °C in presence of 5% CO_2_, and *C. albicans* was cultured in sabouraud dextrose agar (Merck, Germany) in aerobic condition at 37 °C for 24 h, giving a 0.5 McFarland standard.

### Evaluation of antimicrobial effects of acrylic discs containing selected CurNisNps

Antimicrobial effects of selected CurNisNps (acrylic resin containing maximum CurNisNps content with clinically acceptable flexural strength) were evaluated by two different tests:

1. Disc Agar Diffusion test (DAD)
  According to the Clinical Laboratory Standards Institute (CLSI guideline) [33], the CurNisNps solubility, diffusion, and antimicrobial activity in the agar-based medium around acrylic resin discs containing selected CurNisNps were assessed after 0, 15, 30, and 60 days of artificial aging using the DAD test.
2. Biofilm inhabitation test
  The acrylic discs doped with selected concentrations of CurNisNps underwent artificial aging for 0, 15, 30, and 60 days and then were immersed in 48-well microtiter plates containing microbial suspension at a concentration of 0.5 McFarland standard. The microtiter plates were incubated at 37 °C to form the microbial biofilms. After 48 h, the planktonic microorganisms with weak attachments were omitted by rinsing with sterile saline (PBS; pH 7.4) for 1 min. Each specimen was then stained with 0.1% (w/v) crystal violet for 20 min at room temperature and then washed with distilled water two times. The attached biofilm was dissolved by using 200 µL of 95% (v/v) ethanol for 20 min. The dye bound to the biofilm was solubilized in 33% (v/v) acetic acid and the optical density of each well was measured at the wavelength of 570 nm using an automatic microplate reader (Thermo Fisher Scientific, US).

### Evaluation of metabolic activity using XTT reduction assay

The metabolic activity of biofilm-grown *S. mutans* and *C. albicans* cells on acrylic discs doped with selected concentrations of CurNiNps was measured by the reduction of sodium 3-[1-(phenylamino-carbonyl)-3, 4-tetrazolium]-bis (4-methoxy-6-nitro) benzene sulfonic acid hydrate (XTT Kit; Roche Applied Science, Indianapolis, IN, US) based on the manufacturer’s instructions to produce a soluble formazan. The formed microbial biofilms on the acrylic discs were exposed to sonication at 10 W for 20 s and the microbial suspensions were incubated with 250 μL of XTT solution at 37 °C. After 2 h, the absorbance of the supernatant was spectrophotometrically measured using a microplate reader at 490 nm.

### Evaluation of biofilm formation-associated virulence gene expression

For estimation of the expression of virulence factor-related genes in *S. mutans* and *C. albicans*, the biofilms-grown *S. mutans* and *C. albicans* cells on acrylic discs doped with selected concentrations of CurNiNps were separated using ultrasonic waves. The total RNAs were extracted using the super RNA extraction kit (AnaCell, Iran) based on the manufacturer’s instructions. The quantification and qualification of the extracted RNA were measured by a spectrophotometer (Thermo Scientific, USA) and agarose gel electrophoresis, respectively. A high-capacity cDNA reverse transcription kit (Thermo Scientific, USA) was used for cDNA synthesis. The primers and genomic sequences used in this study are listed in Table 1; the 16S rRNA gene and ACT1 were used for normalization. The reaction was carried out by using the Line-Gene K Real-Time PCR System (China) as follows: 95 °C for 3 min, followed by 40 cycles of 95 °C for 15 s, annealing for 30 s at 60 °C, and 72 °C for 30 s, using 10 µL of SYBR® Premix Ex Taq™ II (Tli RNaseH Plus; TaKaRa Bio Inc., Japan), 1 µL of forward primer, 1 µL of reverse primer (both 2.5 pmol), 1 µL of cDNA template (standardized at 50 ng/μL), and 7 μL of distilled water (total 20 µL). The transcription differences were calculated as described [34, 35] and fold changes ≥ 2 were considered biologically significant.

**Table 1:** The primer sequences in this study

Microorganism	Gene	Sequence 5′–3′	Size (pb)	References
*S. mutans*	*gtfB*-F	TGTTGTTACTGCTAATGAAGAA	130	[34]
	*gtfB*-R	GCTACTGATTGTCGTTACTG		
	*16S rRNA*-F	GTGAAATCCCCGGGCTTAAC	217	
	*16S rRNA*-R	ACCGTTTACAGCGTGGACTA		
*C. albicans*	*HWP*-F	GCTCAACTTATTGCTATCGCTTATTACA	67	[35]
	*HWP*-R	GACCGTCTACCTGTGGGACAGT		
	*ACT1*-F	GCTGGTAGAGACTTGACCAACCA	87	
	*ACT1*-R	GACAATTTCTCTTTCAGCACTAGTAGTGA		

bp, base pair

### Statistical analysis

Data were analyzed in the statistical package for social sciences (SPSS) software version 25 by using One-way Analysis of Variance (ANOVA). The Bonferroni post hoc test was applied for comparison between the test groups and *P* < 0.05 was considered statistically significant.

## Results

### Confirmation of synthesized CurNisNps

**Fig. 1:**
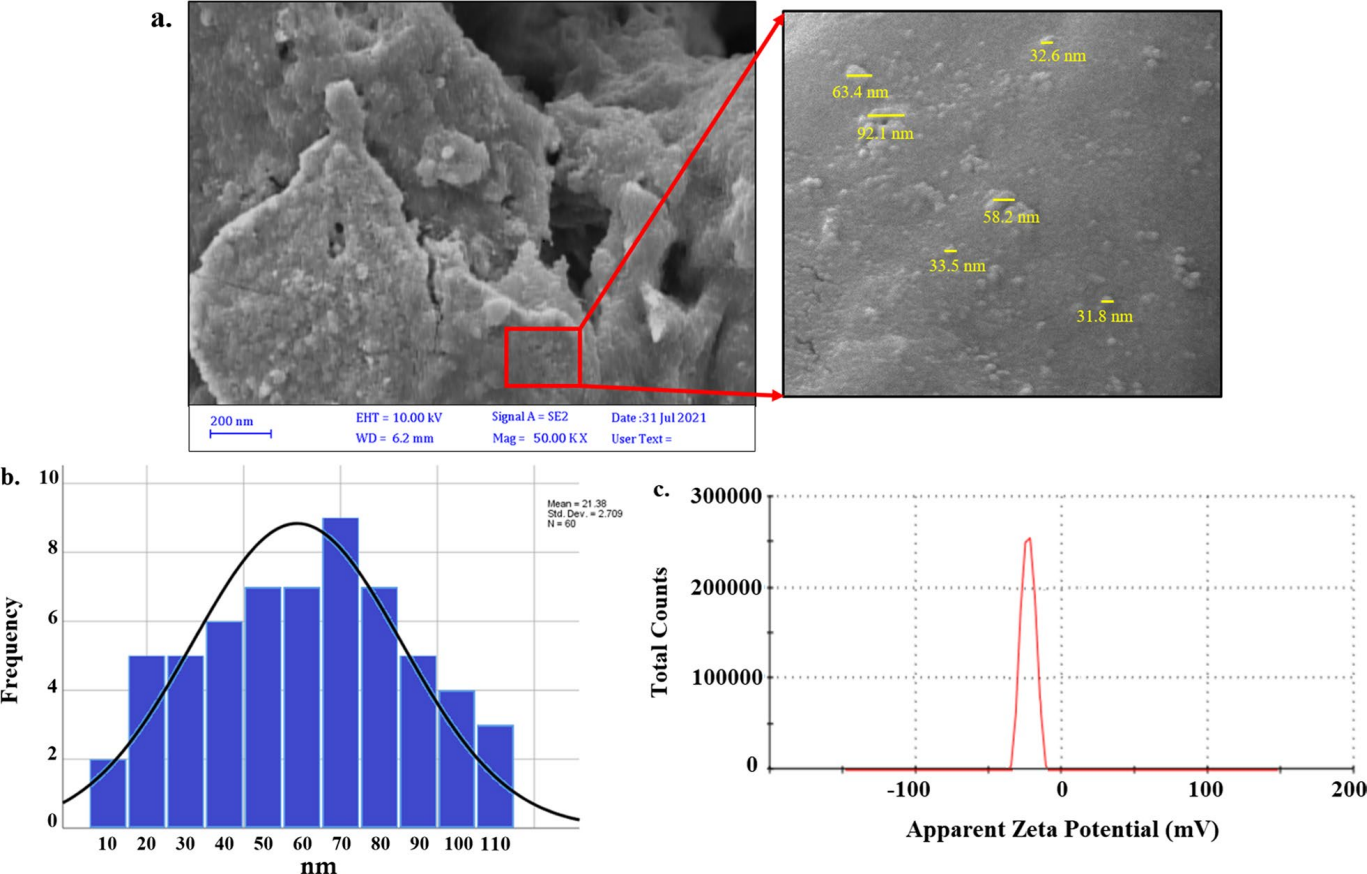
Characterization of synthesized CurNisNps: **a** FESEM image of CurNisNps (Scale bar = 200 nm, Mag = 50.00 KX), **b** average diameter, **c** Zeta potential

FESEM analysis was performed to investigate the surface morphology of CurNisNps and it was shown in Fig. 1a. The surface of CurNisNps appeared to be homogeneous with a spherical shape. According to the results, the average size of CurNisNps was 57.8 ± 17.9 nm (Fig. 1b) and the Zeta potential of CurNisNps was -30.7 ± 4.84 mV (Fig. 1c).

**Fig. 2:**
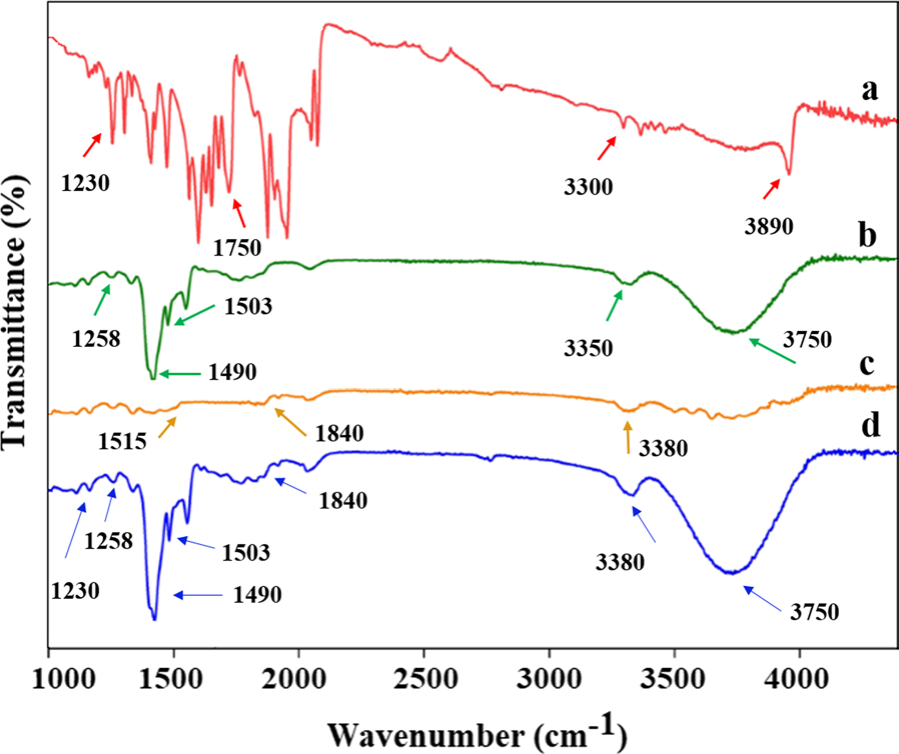
FTIR spectrum of CurNisNps powder: **a**; PLA, **b** Cur, **c** Nis, and **d** CurNisNp

The FTIR spectra of pure PLA, Cur, Nis, and CurNisNps were shown in Fig. 2. In the PLA spectrum, the characteristic peaks are observed between 1230 cm^−1^ and 1750 cm^−1^, which belong to the C=O stretching, and 3300–3890 cm^−1^ for the O–H stretch. Nis has the major characteristics peaks which appeared 1515 cm^−1^, 1840 cm^−1^, and 3380 cm^−1^, which were attributed to the bending of primary amines, amide I, and O–H asymmetrical stretch, respectively. In the Cur spectrum, the peaks at 3350–3750 cm^−1^ characterize the stretching vibrations due to O–H, furthermore, stretching vibration at 1490 cm^−1^ is associated with the aromatic moiety C=C stretching. The peak at 1503 cm^−1^ belongs to the C=O and C=C vibrations and a bending vibration at 1258 cm^−1^ attributed to the phenolic C–O group. As expected, no new peak appeared in CurNisNps confirming that there was no interfacial interaction and chemical bonding between PLA, Cur, and Nis.

### Flexural strength test

The mean and standard deviation of flexural strength values for each group is presented in Table 2. The highest and the lowest flexural strength values were seen in the control group (50.67 ± 1.82 MPa) and in the 10% concentration of CurNisNps group (30.76 ± 3.91 MPa), respectively. The flexural strength values were decreased by increasing the concentration of CurNisNps. According to the results of one-way ANOVA and the Bonferroni post hoc test showed that the significant difference just was observed in the 10% CurNisNps group (*P* < 0.05). Therefore, 5% concentration of CurNisNps was defined as the maximum concentration of CurNisNp that had no statistically significant detrimental effect on flexural strength and was used for antimicrobial tests.

**Table 2:** Mean and standard deviation values of flexural strength in various groups

Concentration of CurNisNps (%)	MPa (mean ± SD)	*P* value
0 (control)	50.67 ± 1.82	–
1	48.85 ± 3.94	0.128
2	45.08 ± 3.33	0.091
5	42.74 ± 4.82	0.058
10	30.76 ± 3.91	0.012

MPa: Megapascal, SD: Standard deviation

### DAD test

The mean size of growth inhabitation zones (GIZs) around acrylic discs at the concentrations of CurNisNps in different time intervals were shown in Table 3. A GIZ was seen around acrylic discs in all time intervals for both test microorganisms and it was larger in the culture of *S. mutans* than the culture of *C. albicans*. The GIZs of PMMA containing CurNisNps against *S. mutans* and *C. albicans* were dose-dependent, while the GIZs of the different concentrations of CurNisNps against test microorganisms were negatively associated with the time, in such a way that it was reduced significantly after 60 days (*P* < 0.05). The orthodontic acrylic resin containing the 5% concentration of CurNisNps effectively restrained *S. mutans* and *C. albicans* growth until 30 days of aging.

**Table 3:** Mean growth inhibition zone size of orthodontic acrylic resin containing selected concentrations of CurNisNps in different time intervals against test microorganisms

Microorganism type	CurNisNps concentrations (%)											
	1				2				5			
	Days											
	1	15	30	60	1	15	30	60	1	15	30	60
	Growth inhibition zone (mm)				Growth inhibition zone (mm)				Growth inhibition zone (mm)			
*S. mutans*	11	10	10	6	13	13	11	10	18	18	16	10
*C. albicans*	9	8	6	4	11	11	9	5	15	14	14	7

### Anti-biofilm activity

Table 4 shows the results of the anti-biofilm test of acrylic resin containing selected concentrations of CurNisNps. Until 30 days of artificial aging, microbial biofilms were not formed on any of the acrylic discs (exception of *C. albicans* biofilm at a concentration of 1% CurNisNps), however, after 60 days of aging, *S. mutans* and *C. albicans* was able to form biofilms on acrylic discs. Modified acrylic resin significantly reduced *S. mutans* and *C. albicans* colony count to 77.52%, and 68.4% after 60 days of aging compared to the control group, respectively (*P* < 0.05).

**Table 4:** Mean optical density of microbial biofilms on orthodontic acrylic resin containing selected concentrations of CurNisNps in different time intervals

Microorganism type	Control	CurNisNps concentrations (%)											
		1				2				3			
		Days											
		1	15	30	60	1	15	30	60	1	15	30	60
	OD (570 nm)	OD (570 nm)				OD (570 nm)				OD (570 nm)			
*S. mutans*	2.99 ± 0.26	NG	NG	NG	1.97 ± 1.7	NG	NG	NG	1.46 ± 1.3	NG	NG	NG	0.68 ± 0.27
*C. albicans*	3.12 ± 0.18	NG	NG	2.38 ± 0.8	3.02 ± 1.1	NG	NG	NG	2.55 ± 1.8	NG	NG	NG	1.09 ± 0.20

NG: No growth

### Anti-metabolic activity of CurNisNps

**Fig. 3:**
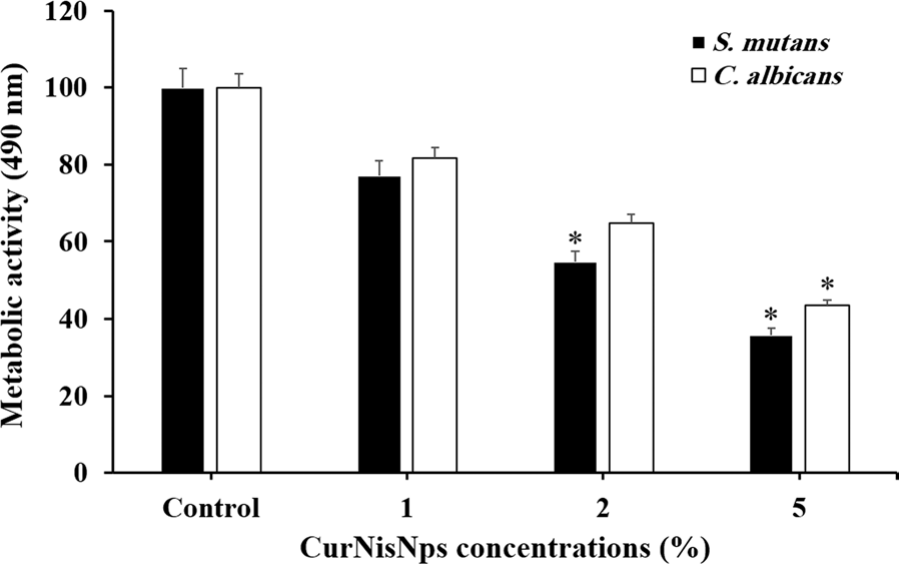
Effect of selected concentrations of CurNisNps on metabolic activity of *S. mutans* and *C. albicans*. *Significantly different from the control group (no treatment), *P* < 0.05

The metabolic activities of *S. mutans* and *C. albicans* biofilms were determined by the XTT assay. As shown in Fig. 3, the orthodontic acrylic resin containing 5% CurNisNps significantly reduced the metabolic activity of *S. mutans* (64.2%) and *C. albicans* (56.5%) when were compared with the control group (*P* < 0.05). Moreover, 2% CurNisNps only remarkably decreased the *S. mutans* metabolic activity to 45.2% (*P* < 0.05). No significant reduction in metabolic activity of *S. mutans* and *C. albicans* was determined in the orthodontic acrylic resin containing 1% CurNisNps (*P* > 0.05).

### Expression of virulence genes

**Fig. 4:**
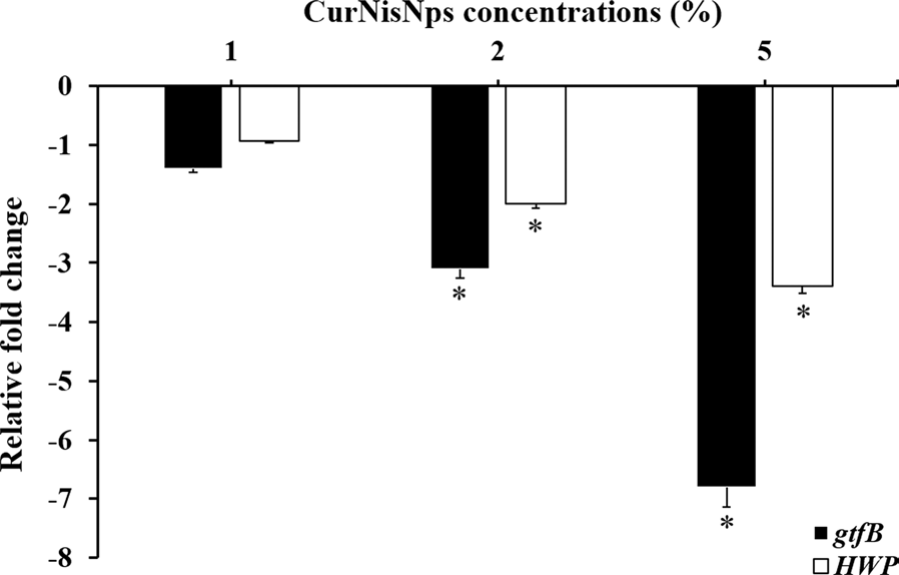
The relative fold change in expression levels of biofilm formation-associated virulence genes. *Significantly different from the control group (no treatment), *P* < 0.05

According to the results, changes in gene expression are dose-dependent. As shown in Fig. 4, the gene expression profiling of *gtfB* was downregulated in *S. mutans* cells, with the greatest reduction seen for 5% CurNisNps, which was ~ 3.4-fold higher than *HWP* gene in *C. albicans*. There was no remarkable difference in the expression of *gtfB* and *HWP* genes under treatment conditions with 1% CurNisNps (*P* > 0.05).

## Discussion

Removable orthodontic acrylic appliances are routinely used owing to their many benefits such as easy and simple fabrication, affordable price, favorable esthetic, and low chairside time. The cold-cure acrylic resins which are used for manufacturing of these appliances suffer from more porosity in their structure than heat-cure ones and as the palatal surface of them are not polished to gain maximum soft tissue adaptation, the rough surface of them could be a place for accumulation of opportunist microorganisms and biofilm formation. Shifting normal oral flora to more pathogenic flora and higher incidence of enamel demineralization and gingival inflammation has been reported after long-term use of these appliances [5, 6]. Diverse nanoparticles have been added to orthodontic acrylic resins to give them self-sterilizing features and to reduce biofilm formation around them [13–16]. Since these nanoparticles are mainly derived from metallic ions, some worries exist regarding their possible harmful biological and environmental effects [17]. In this study, CurNisNps as an organic natural substance with antimicrobial properties were added into orthodontic acrylic resin. Since fracturing of acrylic appliances because of their poor mechanical characteristics has been a clinical concern [26], initially, the flexural strength of this new synthetic compound was assessed to determine the suitable concentration of the modifier. Then, the antimicrobial properties of acrylic resin containing the optimal concentration of CurNisNps against *S. mutans* and *C. albicans* were investigated.

Shahabi et al. [36] assessed the effect of the addition of chitosan nanoparticles, biocompatible and organic polysaccharides with antimicrobial activity, to cold-cure acrylic orthodontic acrylic resin on its flexural strength. They observed with increasing concentration of chitosan NPs up to 1% (w/w), the flexural strength did not reduce significantly, however in 2% and 4% concentration of chitosan nanoparticles, flexural strength reduced significantly compared to the control group. Pourhajibagher et al. [32] investigated the impact of the incorporation of *Ulva lactuca*, green macro-algae with proven antimicrobial activity, into cold-cure autopolymerizing orthodontic acrylic resin on its flexural strength. Adding up to 1% (w/w) concentration of *U. lactuca* to acrylic resin did not significantly affect its flexural strength whereas incorporating higher concentration (5% and 10%) adversely affected its flexural strength. In contrast, Ajami et al. [37] reported that incorporating various concentrations (0.4, 0.8, and 1.6%) of *Galla chinensis* extract (GCE), traditional Chinese medicine with anti-cariogenic properties, into orthodontic acrylic resin, increases its flexural strength significantly compared to control group. They did not observe significant differences in flexural strength values between study groups containing different concentrations of GCE. These findings imply that the type of organic antimicrobial compound added to the orthodontic acrylic resin and its concentration both play role in the flexural strength of the final compound. The organic modifier could disrupt the integrity of acrylic resin and detrimentally affects its flexural strength or on the contrary could act as a cross-linker and enhances its flexural strength; therefore, the optimal concentration of any modifier should be confirmed before clinical use. The results of the current study showed that adding CurNisNps into cold-cure orthodontic acrylic resin decreased its flexural strength. This adverse effect was not remarkable at a concentration of 1, 2, and 5% (w/w), however, increasing CurNisNps concentration to 10% (w/w), significantly reduced the flexural strength of the final product.

Regarding the DAD test, Arab et al. [38] did not observe growth inhibition zones around orthodontic acrylic disks containing different concentrations (0.5, 1, and 2%) of propolis nanoparticles. Pourhajibagher et al. [39] did not report growth inhibition zones around acrylic resin discs containing various concentrations (0.5, 1, and 2%) of *Undaria pinnatifida* a brown seaweed microalga, too. In contrast, Ajami et al. [37] reported dose-dependent GIZs around orthodontic acrylic resin samples containing diverse concentrations (0.4, 0.8, and 1.6%) of GCE. Pourhajibagher et al. [32] observed dose-related growth inhibition zones around acrylic resin discs containing *U. lactuca* in the DAD test, too. The results of our study showed GIZs around acrylic disks fabricated from modified orthodontic acrylic resin that was contained 5% (w/w) concentration of CurNisNps in the culture of *S. mutans* and *C. albicans*, both. This finding implies solubility and diffusion of CurNisNps in the agar-based medium around acrylic resin discs, so CurNisNps have a contact antimicrobial effect and is an appropriate modifier to enhance antimicrobial characteristics of orthodontic acrylic resin.

The results of the present study showed remarkable anti-biofilm activity of orthodontic acrylic resin containing 5% (w/w) of CurNisNps against *S. mutans* and *C. albicans* until 60 days of follow-up. The results of crystal violet assay showed that *S. mutans* and *C. albicans* did not grow on the surface of the modified acrylic resin containing 5% of CurNisNps until 30 days of follow-up and the levels of *S. mutans* and *C. albicans* reduced by 77.52% and 64.8% at 60 days of follow-up, respectively. In line with this study, Pourhajibagher et al. [39] reported dose-dependent anti-biofilm activity of acrylic resin doped with *U. pinnatifida* against *S. mutans*. They observed that acrylic resin containing 0.5, 1, and 2% concentrations of *U. pinnatifida* after photodynamic therapy could decrease *S. mutans* colony count to 79%, 97%, and 99%, respectively. The results of the current study are consistent with a recent report [32], in which the addition of *U. lactuca* enhanced the antimicrobial activity of orthodontic acrylic resin in dose-dependent manner. Arab et al. [38], reported anti-biofilm activity of orthodontic acrylic resin doped with 1% and 5% concentration of propolis nanoparticles after 1 and 3 days of follow-up against *S. mutnas*, *C. albicans*, *S. sanguinis*, and *Lactobacillus acidophilus*, however, the acrylic resin containing 0.5% of propolis nanoparticles did not show significant anti-biofilm effect. Ajami et al. [37] reported the microbiocidal impact of orthodontic acrylic resin containing different concentrations of GCE against biofilm of *S. mutans* until 1 day of follow-up, but there was no difference in reduction of colony count among groups with 0.4, 0.8, and 1.6% concentrations of GCE. According to flexural strength and anti-biofilm tests, an orthodontic acrylic resin containing 5% concentration of CurNisNps has considerable anti-biofilm activity against *S. mutans* and *C. albicans* until 60 days of follow-up.

According to the findings of this study, CurNisNps could significantly downregulate the expression levels of *gtfB* and *HWP*, as the virulence factors in *S. mutans* and *C. albicans*, respectively. It appears that, following the reduction of the expression of these virulence genes, the biofilm formation ability in *S. mutans* and *C. albicans* also decreases. Our results also suggested that when CurNisNps was used at high concentration, the metabolic activities of these microorganisms, followed by the ability of biofilms growth were decreased. Further studies are needed to better understand the metabolic pathways involved in pathogenic and biological-associated properties of *Streptococcus* spp. and *Candida* spp.

The limitation of the present study is an assessment of anti-biofilm activity against only *S. mutans* and *C. albicans* species, since various microorganisms play role in the formation of cariogenic biofilm in the oral cavity, it will be beneficial to investigate the microbiocidal and anti-biofilm activity of orthodontic acrylic resin containing CurNisNps against multispecies biofilm-generating microbiota. There is also a need for more *in-vitro* with a longer follow-up period, animal models study to simulate the oral cavity environment, and clinical trial to evaluate the anti-biofilm and mechanical properties of this new synthetic compound.

## Conclusion

According to the results, the modified acrylic resin containing 5% CurNisNps significantly reduced the microbial population and metabolic activity of *S. mutans* and *C. albicans* after 60 days of aging. Until 30 days of artificial aging, *S. mutans* and *C. albicans* microbial biofilms were not formed on the acrylic discs containing 5% CurNisNps. The biofilm formation-associated virulence gene expression profiling of *gtfB* and *HWP* was downregulated in *S. mutans* and *C. albicans* cells, respectively following treatment with 5% CurNisNps. Our study showed that 5% (w/w) of CurNisNps can serve as an excellent orthodontic acrylic resin additive against *S. mutans* and *C. albicans* biofilm with potential clinical applications for the prevention of dental caries, periodontal diseases, and candidiasis in removable orthodontic treatment without adverse effects on its mechanical property.

## Data Availability

All data of this manuscript are included in the manuscript. All figures are original images and have been used for the first time in this study. Any additional information required will be provided by communicating with the corresponding author via the official mail: abahador@sina.tums.ac.ir.

## Abbreviations

CurNisNps: Curcumin-Nisin-poly(l-lactic acid) nanoparticle (CurNisNps)
MPa: Megapascal
w/w: Weight/Weight
